# Iron acquisition strategies in pseudomonads: mechanisms, ecology, and evolution

**DOI:** 10.1007/s10534-022-00480-8

**Published:** 2022-12-12

**Authors:** Rolf Kümmerli

**Affiliations:** Department of Quantitative Biomedicine, University of Zürich, Winterthurerstrasse 190, 8057 Zurich, Switzerland

**Keywords:** Ferrous iron importer, Siderophores, Citrate, Heme, Siderophore exploitation and competition, Diversifying selection

## Abstract

Iron is important for bacterial growth and survival, as it is a common co-factor in essential enzymes. Although iron is very abundant in the earth crust, its bioavailability is low in most habitats because ferric iron is largely insoluble under aerobic conditions and at neutral pH. Consequently, bacteria have evolved a plethora of mechanisms to solubilize and acquire iron from environmental and host stocks. In this review, I focus on *Pseudomonas* spp. and first present the main iron uptake mechanisms of this taxa, which involve the direct uptake of ferrous iron via importers, the production of iron-chelating siderophores, the exploitation of siderophores produced by other microbial species, and the use of iron-chelating compounds produced by plants and animals. In the second part of this review, I elaborate on how these mechanisms affect interactions between bacteria in microbial communities, and between bacteria and their hosts. This is important because *Pseudomonas* spp. live in diverse communities and certain iron-uptake strategies might have evolved not only to acquire this essential nutrient, but also to gain relative advantages over competitors in the race for iron. Thus, an integrative understanding of the mechanisms of iron acquisition and the eco-evolutionary dynamics they drive at the community level might prove most useful to understand why *Pseudomonas* spp., in particular, and many other bacterial species, in general, have evolved such diverse iron uptake repertoires.

## Introduction

The first research report on iron-acquisition strategies in *Pseudomonas* spp. goes back to 1892 (Gessard 1892), in which the putative function of a fluorescent pigment (later described as the siderophore pyoverdine) was examined. While initial work was carried out with various *P. fluorescens* spp. (Turfitt 1936; Meyer and Abdallah 1978; Meyer and Hornsperger 1978; Teintze et al. 1981), *P. aeruginosa* soon became the major focus of research with regard to iron-uptake mechanisms (Totter and Moseley 1953; Cox and Graham 1979; Cox et al. 1981; Wendenbaum et al. 1983). The primary reason for this is certainly that *P. aeruginosa* is an opportunistic human pathogen with many of the iron-uptake strategies being involved with virulence (Messenger and Barclay 1983; Ankenbauer et al. 1985; Meyer et al. 1996; Takase et al. 2000; Konings et al. 2013; Granato et al. 2016; Winstanley et al. 2016). The link to pathogenicity has fueled interests in understanding the biochemical and molecular basis of iron acquisition in this species and its interactions with hosts (Harrison et al. 2006; Kirienko et al. 2013; Newman et al. 2017; Weigert et al. 2017; Kang et al. 2018). Only at a later stage, interest in other *Pseudomonas* spp. was renewed and researchers started to compare iron acquisition mechanisms across species and strains (Cornelis and Matthijs 2002; Ravel and Cornelis 2003; Smith et al. 2005; Meyer et al. 2008; Cornelis 2010). This body of work revealed tremendous diversity and variation in iron uptake mechanisms across species and consequently spurred interest in ecological and evolutionary questions (Griffin et al. 2004; Smith et al. 2005; Cordero et al. 2012; Lee et al. 2012; Bruce et al. 2017; Butaitė et al. 2017; Kramer et al. 2020). Why are there so many different ways to obtain iron? What were the abiotic and biotic conditions that favored the evolution and maintenance of such diverse iron-uptake repertoires? While these questions are still under investigation, it seems timely to bring the knowledge from biochemistry, molecular biology, ecology and evolution together in order to understand iron acquisition strategies in *Pseudomonas* spp. at a more integrative level. This is the aim of this review. Important to note is that there are many excellent reviews on the molecular mechanisms of iron acquisition and its regulation in pseudomonads (Vasil and Ochsner 1999; Poole and McKay 2003; Visca et al. 2007; Cornelis et al. 2009; Mossialos and Amoutzias 2009; Cornelis 2010; Youard et al. 2011; Schalk and Guillon 2013; Lau et al. 2016; Rivera 2017; Ringel and Brüser 2018; Schalk et al. 2020). While I cover essential mechanistic aspects in this review, I leave out many of the molecular details on regulatory, synthesis and iron uptake machineries. I do not do this out of ignorance, but because the molecular details have been extensively presented in these previous reviews, which allows me to discuss iron acquisition at a more integrative level.

### The many ways to take up iron in ***Pseudomonas*** spp.

Iron is an essential co-factor in numerous enzymes in many bacterial taxa (Neilands 1974; Schröder et al. 2003). Iron is particularly potent as co-factor because of its ability to transform between the ferrous (Fe^2+^) and the ferric (Fe^3+^) oxidation stage and thereby facilitate redox reactions (Morgan and Lahav 2007; Sepulveda Cisternas et al. 2018). Iron fulfills this function in a wide set of enzymes, including cytochromes, catalases, hydrogenases and superoxide dismutases (Messenger and Barclay 1983). While the role of iron for microbial metabolism is undisputed, a more challenging question is how bacteria can access iron from environmental sources. The problem is that ferric iron is insoluble under many conditions (Morgan and Lahav 2007). It either precipitates as iron hydroxide (Fe(OH)_2_ or Fe(OH)_3_) in aqueous solutions, or occurs in the form of minerals such as iron-oxide (Fe_2_O_3_), magnetite (Fe_3_O_4_) and pyrite (FeS_2_) (Schröder et al. 2003). Low bioavailability of iron is particularly strong at neutral pH and aerobic conditions, as prevailing in many natural habitats (Lovley and Philipps 1986; Boyd and Ellwood 2010; Colombo et al. 2013). In the first part of the review, I will provide an overview on how bacteria have overcome this problem and have evolved a broad range of mechanisms to secure iron for metabolism. I focus on *Pseudomonas*, as it is probably the best studied genus in the context of iron acquisition. But the interested reader can find detailed information on the iron-acquisition strategies of other bacterial taxa elsewhere (Guerinot 1994; Ratledge and Dover 2000; Andrews et al. 2003; Wandersman and Delepelaire 2004; Miethke and Marahiel 2007; Sandy and Butler 2009; Hider and Kong 2010; Braun and Hantke 2011; Frawley and Fang 2014; Kümmerli et al. 2014).

#### Direct uptake of iron through membrane-embedded importers

**Fig. 1:**
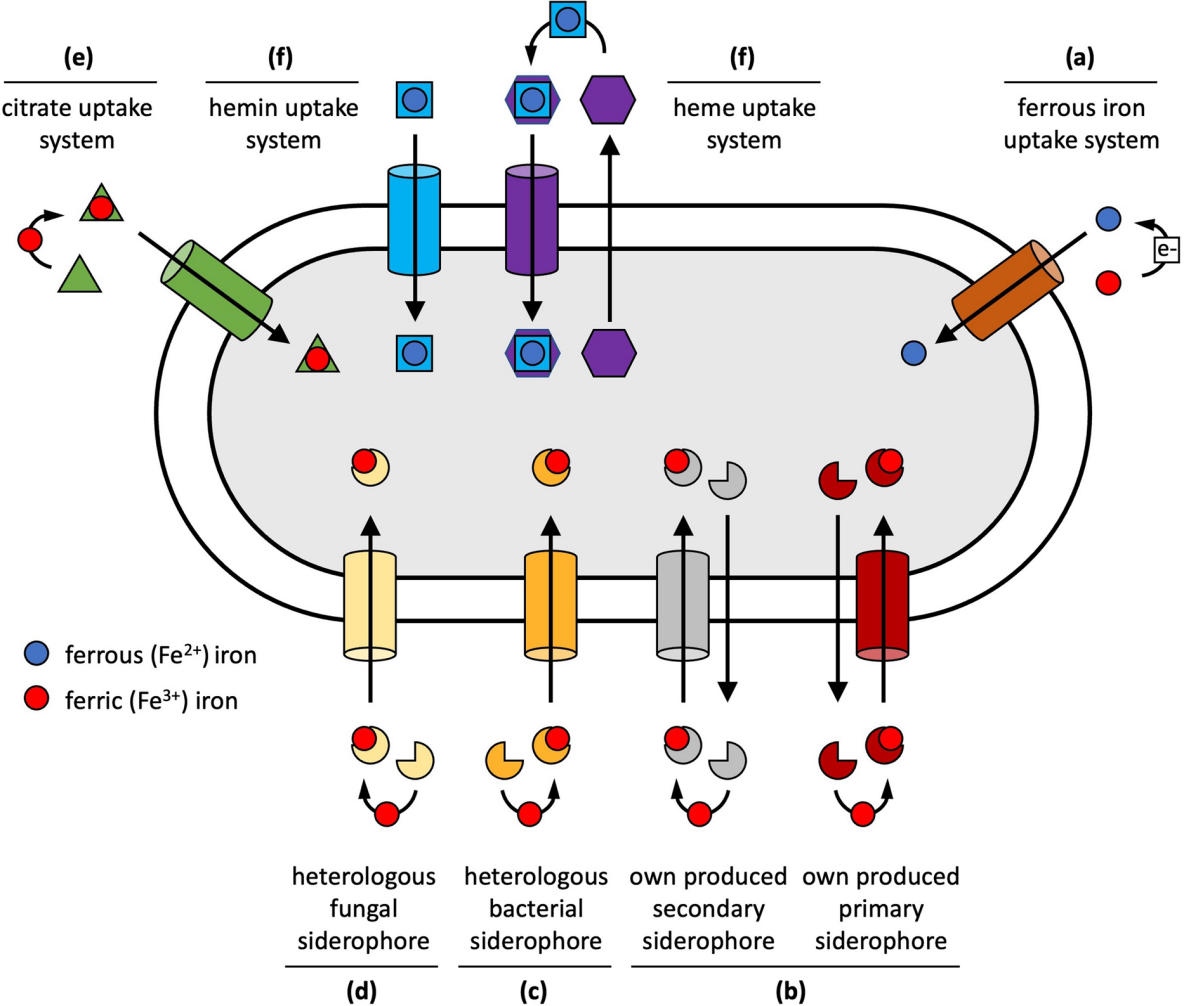
Schematic of iron acquisition systems in *Pseudomonas* spp. All iron transporters are shown as cylinders for reasons of simplicity. The molecular complexity of the various uptake systems is covered elsewhere (Cornelis and Dingemans 2013; Schalk and Cunrath 2016). **a** Ferrous iron permeases (like FeoB and EfeU) can directly take up Fe^2+^ without the need of a carrier. In environments, where ferric (Fe^3+^, red circle) iron prevails, a reduction step to Fe^2+^ (blue circle), for example through phenazines, is required. **b** Siderophores (three-quarter circles) are secondary metabolites that are secreted in the environment to scavenge iron. Most *Pseudomonas* spp. produce pyoverdine as their primary siderophore with strong iron affinity (Fig. 2), and a variety of secondary siderophores with lower iron affinity (Fig. 3). The ferri-siderophore complexes are recognized and internalized via outer membrane-embedded TonB-dependent transporters. **c** Transporters for the uptake of heterologous ferri-siderophores (e.g., enterobactin, desferrioxamines) produced by other bacterial species. **d** Transporters for the uptake of heterologous fungal ferri-siderophores (e.g. ferrichrome). **e** Transporters for the uptake of ferri-citrate (green triangle), a metabolite and iron chelator exuded from plant roots. **f** Transporters for the uptake of the iron-containing heme group (blue square). The Phu-system and the HxuA system (on the left) can directly take up the heme group. The Has-system (on the right) relies on the secretion of a hemophore protein (purple hexagon) for heme scavenging

The most straightforward solution for iron acquisition matches the one for general nutrient uptake pathways, which typically operate via membrane-embedded importer systems (Fig. 1a). In this context, *Pseudomonas* spp. including *P. aeruginosa* possess the Feo system, consisting of an iron permease FeoB and the cytoplasmic protein FeoA that allow the uptake of solubilized ferrous iron (Cartron et al. 2006; Marshall et al. 2009; Seyedmohammad et al. 2014; Lau et al. 2016). Moreover, a second ferrous iron importer system (EfeUOB), first described in *Escherichia coli* (Grosse et al. 2006; Cao et al. 2007), has now also be found in certain *Pseudomonas* species, e.g. *P. syringae* (Rajasekaran et al. 2022). This system consists of the permease EfeU and two periplasmic proteins EfeO and EfeB. Ferrous iron permeases and their associated proteins could allow efficient iron uptake in habitats characterized by low pH and/or low oxygen availability, which means under conditions where iron solubility is increased and ferrous iron is not instantly oxidized to ferric iron (Cao et al. 2007; Lau et al. 2016). The situation looks different at neutral pH and aerobic conditions. Here, ferrous iron permeases can still be beneficial but only when working in concert with iron reducing agents. For example, phenazines are redox-active molecules secreted by many *Pseudomonas* spp. (Mavrodi et al. 2013). They are known to spontaneously reduce ferric to ferrous iron in the environment and thereby fueling iron uptake via permeases (Cox 1986; Wang et al. 2011). More recently, it was discovered that catecholamine neurotransmitter can also reduce iron and spur ferrous iron uptake via permeases (Perraud et al. 2022). However, permease-mediated iron acquisition rates might be lower compared to iron uptake rates mediated by siderophores (presented in the next section), an effect that has been demonstrated in *Burkholderia cenocepacia* (Mathew et al. 2014). From an evolutionary perspective, it is assumed that ferrous iron import systems represent an ancestral mechanism of iron uptake, as they occur in a conserved manner across many microbial taxa (Hantke 2003; Cartron et al. 2006; Lau et al. 2016).

#### Iron acquisition through siderophores

**Fig. 2:**
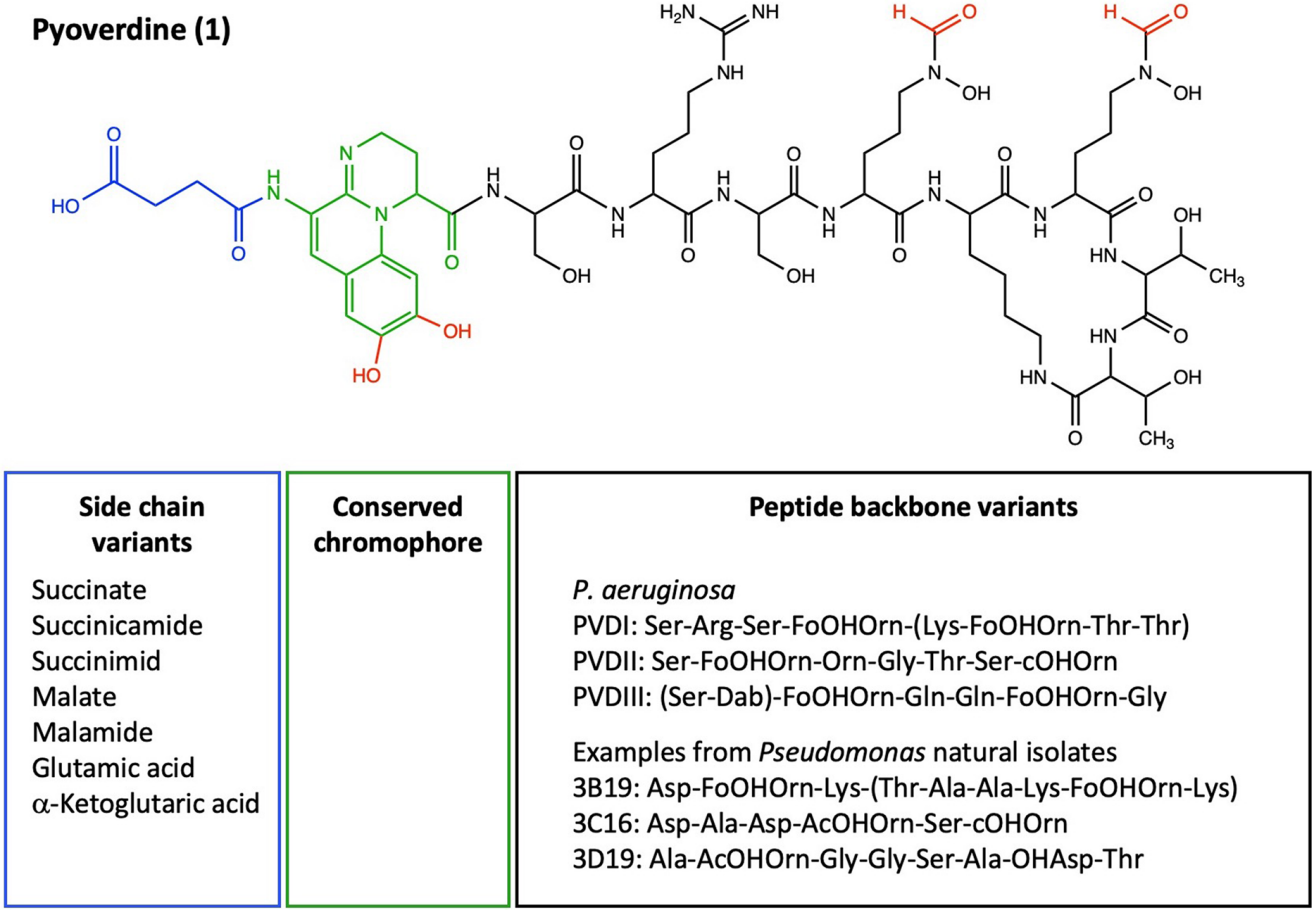
Pyoverdine and its structural diversity among *Pseudomonas* spp**.** The primary siderophore pyoverdine consists of a conserved chromophore (green), a strain-specific peptide backbone (black) with a variable number of amino acids, and a variable set of side chains (blue). The functional groups marked in red are involved in iron chelation. The black box shows examples of peptide backbone variation found among *P. aeruginosa* strains (Schalk et al. 2020) and among co-isolated natural strains (Rehm et al. 2022). The blue box shows a list of side chain variants found among co-isolated natural strains (Rehm et al. 2022)

Another and very common way of how bacteria bring iron into solution operates via siderophores (Fig. 1b). Siderophores are secondary metabolites and constitute a class of structurally different molecules that all have the capacity to bind iron (Hider and Kong 2010). A common characteristic of fluorescent *Pseudomonas* spp. is that they produce pyoverdine (1) as their primary siderophore (Meyer 2000; Meyer et al. 2008). Primary in this context means that pyoverdine is the siderophore with the highest iron affinity known among the ones examined in this genus. In addition to pyoverdine, many species also produce other, so called secondary siderophores with lower affinity for iron (Cornelis and Matthijs 2002; Cornelis 2010). I will touch upon the role of these secondary siderophores below. Let us focus on pyoverdine first. Pyoverdine is produced via non-ribosomal peptide synthesis (Visca et al. 2007; Schalk et al. 2020). This means that there is no genetic code for pyoverdine, but a series of genes that encode enzymes that synthetize pyoverdine from biochemical building blocks in the cytosol. Strictly speaking, pyoverdine stands for a class of molecules that can vary between species and strains (Fig. 2) (Meyer 2000; Ravel and Cornelis 2003; Meyer et al. 2008). The molecule consists of three parts: (i) a conserved chromophore that makes this molecule yellow-green fluorescent, (ii) a variable peptide backbone comprising 6 to 12 amino acids, and (iii) a variable side chain (Visca et al. 2007; Schalk et al. 2020). A single strain produces pyoverdine molecules that have always the same peptide backbone but can vary in their side chains. More diversity comes in at the community level, where closely related strains and species can produce pyoverdine types that also differ in their peptide backbone (Smith et al. 2005; Butaitė et al. 2017; Rehm et al. 2022).

**Fig. 3:**
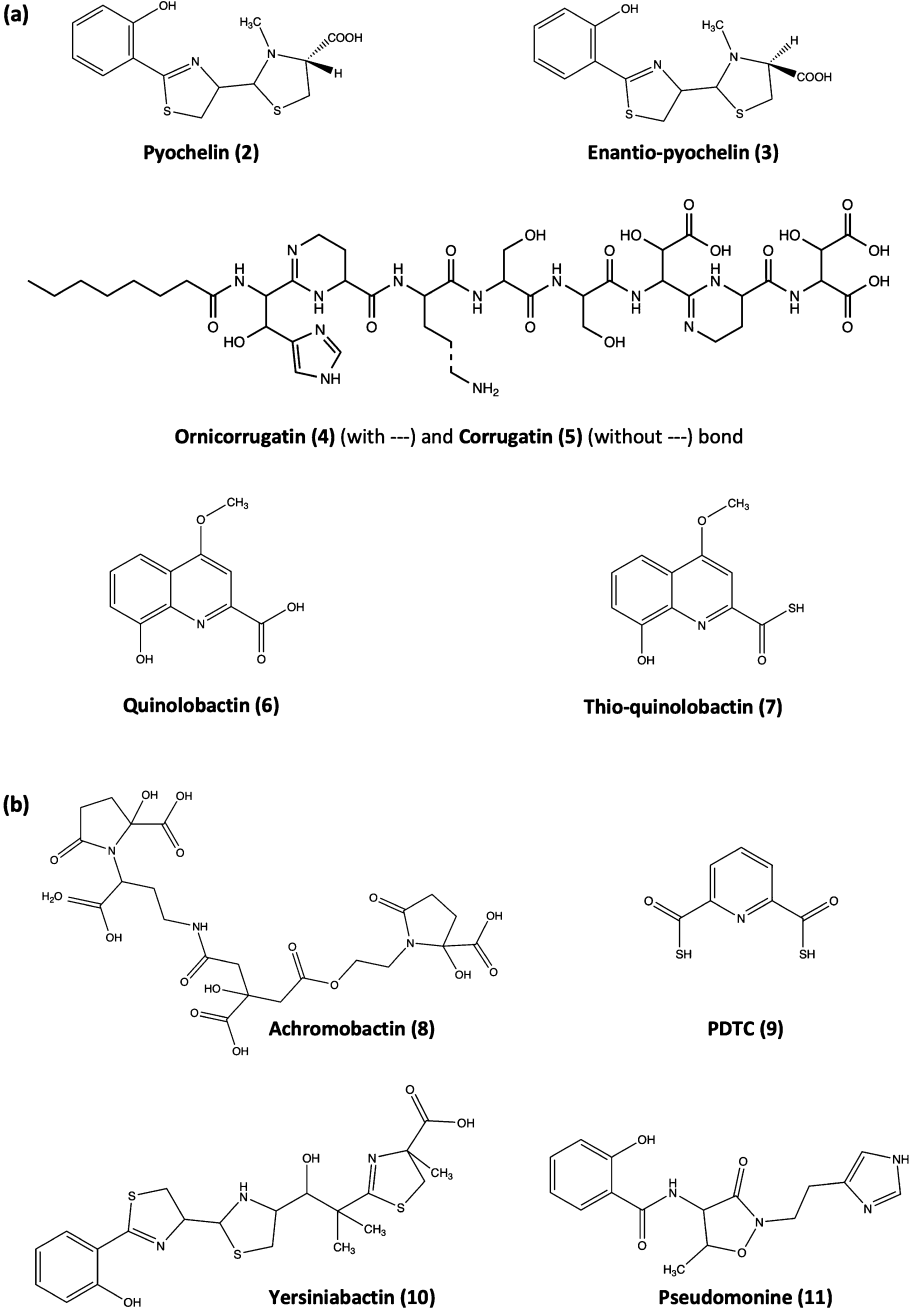
Currently known secondary siderophores in *Pseudomonas* spp. (Cornelis 2010). **a** Secondary siderophores for which structural variants have been described: pyochelin (2) vs. enantio-pyochelin (3), ornicorrugatin (4) vs. corrugatin (5), quinolobactin (6) vs. thio-quinolobactin (7). Some of these variants—pyochelin vs. enantio-pyochelin—are known to confer specificity with regard to the uptake of the iron-loaded siderophores. **b** Secondary siderophores for which a single structural variant exists: achromobactin (8), PDTC (9), yersiniabactin (10), pseudomonine (11)

As mentioned above, many *Pseudomonas* spp. possess synthesis clusters for one or multiple secondary siderophores in their genome. A comparison across strains and species yielded the following secondary siderophores (Cornelis 2010) (Fig. 3): (2) pyochelin—*P. aeruginosa* (Cox and Graham 1979; Cox et al. 1981; Heinrichs et al. 1991; Michel et al. 2005); (3) enantio-pyochelin—*P. protegens* CHAO (Youard et al. 2007; Youard et al. 2011); (4) quinolobactin and (5) thio-quinolobactin—*P. fluorescens* (Mossialos et al. 2000; Matthijs et al. 2007); (6) ornicorrugatin and (7) corrugatin—*P. corrugata* and *P. fluorescens* (Risse et al. 1998; Matthijs et al. 2008); (8) achromobactin—*P. syringae* (Berti and Thomas 2009); (9) PDTC (pyridine-2,6-bis(thiocarboxylic acid))—*P. putida* (Lewis et al. 2004; Leach and Lewis 2006); (10) yersiniabactin—*P. syringae* (Jones et al. 2007); and (11) pseudomonine—*P. fluorescens* and *P. entomophila* (Mercado-Blanco et al. 2001; Matthijs et al. 2009). As pyoverdine, these secondary siderophores are produced by non-ribosomal peptide synthesis (Crosa and Walsh 2002), and similar to pyoverdine, certain secondary siderophores also occur in different chemical variants (Fig. 3a). For others, only a single variant has been described so far (Fig. 3b). Secondary siderophores are common in bacteria, not only in the genus *Pseudomonas*, but also in many other taxa like *Burkholderia* (Thomas 2007) and *Vibrio* (Lemos et al. 2010; Cordero et al. 2012). Often, there is regulatory linkage between the synthesis of the primary and the secondary siderophore within a species, in the sense that the production of the primary siderophore generally suppresses or reduces the synthesis of the secondary siderophore (Cornelis 2010; Lemos et al. 2010; Dumas et al. 2013; Tyrrell et al. 2015; Sathe et al. 2019).

Bringing iron into solution is only half of the battle. Uptake systems are required to bring the solubilized siderophore-bound iron into the cell. This part of the iron acquisition operates via cognate TonB-dependent transporters (often also referred to as receptors), which are embedded into the outer membrane (Andrews et al. 2003; Visca et al. 2007; Youard et al. 2011; Schalk and Guillon 2013). While the transporter itself is responsible for the recognition and internalization of the ferri-siderophore complex into the periplasm, the TonB complex generates the energy required for the uptake. For ferri-pyoverdines, the transporter is FpvA (Folschweiller et al. 2000; Cobessi et al. 2005). As for the pyoverdine molecules themselves, FpvA actually constitutes a family of transporters, whereby each species and strain produces a variant of the transporter that has high specificity for recognizing and taking up the self-produced (ferri-)pyoverdine (De Chial et al. 2003; Greenwald et al. 2009). Detailed knowledge is further available for the uptake of ferri-pyochelin and ferri-enantio-pyochelin, also involving TonB-dependent transporters (Michel et al. 2005; Michel et al. 2007; Braud et al. 2009; Youard et al. 2011; Cunrath et al. 2015). Pyochelin and enantio-pyochelin are identical molecules in terms of their chemical composition and iron-chelation properties but differ in their chirality (Fig. 3a). While *P. aeruginosa* PAO1 produces pyochelin, *P. protegens* CHA0 makes enantio-pyochelin. Intriguingly, the two TonB-dependent transporters FptA and FetA are highly selective for ferri-pyochelin and ferri-enantio-pyochelin, respectively (Michel et al. 2005; Michel et al. 2007; Braud et al. 2009; Youard et al. 2011; Cunrath et al. 2015), followed by an additional selective step at the inner membrane (Reimmann 2012). Figure 3a shows that other secondary siderophores also occur in two forms: corrugatin vs. ornicorrugatin and quinolobactin vs. thioquinolobactin. Whether the chemical variation in these molecules also results in specificity, as shown for the pyochelin variants, remains to be tested.

#### Tapping sources of non-self-produced iron chelators

*Pseudomonas* spp. are known for their versatile lifestyles and their ability to thrive in many different environments (Silby et al. 2011; Moradali et al. 2017). Moreover, they are often members of taxonomically rich microbial consortia and associate with hosts including plants and animals (Silby et al. 2011). Since all organisms must overcome the challenge of iron acquisition, we can expect that *Pseudomonas* spp. are exposed to a rich repertoire of iron-chelating compounds, including non-self-produced (heterologous) siderophores secreted by other consortia members. Consistent with this hypothesis, *Pseudomonas* spp. possess a diverse set of strategies to tap sources of non-self-produced iron chelators.

The first strategy relies on the uptake of heterologous bacterial (ferri-)siderophores via the expression of respective transporters (Fig. 1c). For example, the genes *pirA* and *pfeA* in *P. aeruginosa* encode transporters for enterobactin uptake, a high-iron affinity siderophore produced by Enterobacteriaceae spp. like *E. coli* and *Salmonella typhimurium* (Dean and Poole 1993; Ghysels et al. 2005; Moynie et al. 2019). PfeA can further take up two additional (ferri-)siderophores, azotochelin and protochelin (Moynie et al. 2019). *P. aeruginosa* further possesses the gene *chtA*, encoding the transporter for the uptake of a suite of structurally similar (ferri-)siderophores including aerobactin (produced by *E. coli* and *Aerobacter aerogenes*) rhizobactin 1021 (*Rhizobium meliloti*), and schizokinen (*Bacillus megaterium* and *Ralstonia solanacearum*) (Cuiv et al. 2006). *P. aeruginosa* also has the transporters FvbA for vibriobactin (Elias et al. 2011), FemA for (carboxy-)mycobactin (Llamas et al. 2008), and FoxA for desferrioxamines (Llamas et al. 2006) uptake, a suite of siderophores produced by a diverse set of species including *Vibrio* spp., mycobacteria, *Streptomyces* ssp., *Erwinia amylovora*. This demonstrates that *P. aeruginosa* is able to use (ferri-)siderophores from both animal/human pathogens and environmental bacteria. While transporter diversity is well-studied for *P. aeruginosa*, diversity also seems to be common among environmental *Pseudomonas* spp. (Ye et al. 2014; Galet et al. 2015).

The second strategy is similar to the first one but relies on the uptake of fungal (ferri-)siderophores via the expression of the respective transporters (Fig. 1d). Once more, detailed knowledge is available for *P. aeruginosa* that possesses the gene *fuiA*, coding for the transporter involved in the uptake of ferrichrome (Llamas et al. 2006; Hannauer et al. 2010), a common fungal siderophore produced by members of the genus *Aspergillus*, *Ustilago* and *Penicillium* (Renshaw et al. 2002; Haas et al. 2008). Orthologues of the ferrichrome transporter gene have also been found in environmental pseudomonas isolates (Ye et al. 2014). Important to note is that the ferrichrome transporter was first described for *E. coli* among bacteria (Coulton et al. 1983) where it is annotated as FhuA.

The third strategy relies on the uptake of iron bound to plant iron carriers. To overcome iron limitation, monocotyledon plants secrete phytosiderophores, like mugineic acid, avenic acid, and distichonic acid (Crowley et al. 1991; Hider and Kong 2010; Ahmed and Holmstrom 2014). There is little work on whether bacteria in general and *Pseudomonas* spp. in particular can directly take up iron-phytosiderophore complexes. It would not be surprising if they could do so. Recent transcriptomic data on the interaction between *P. fluorescence* SBW25 and *Brachypodium distachyon* indeed support this notion, although the exact mechanism of phytosiderophore uptake remains to be elucidated (Boiteau et al. 2021). On the other hand, one could also argue that there is little need for bacteria to tap phytosiderophores as iron source, because of the iron chelating properties of organic acids (e.g. oxalate, malate, citrate) exuded from plant roots. Citrate seems to play a major role in this context. While citrate is secreted by plants to improve phosphate mobilization (Gerke 2015) and mediate heavy metal detoxification (Gupta et al. 2013), it also acts as a strong iron chelator forming a ferric-dicitrate complex (Crowley et al. 1991). *P. aeruginosa* possesses the FecA transporter to import the ferric-dicitrate complex (Fig. 1e) (Marshall et al. 2009). The same transporter was also found in other bacterial taxa (Lin et al. 1999; Brown and Holden 2002), suggesting that it is a common and conserved iron-uptake mechanism. Moreover, *P. aeruginosa* seems also able to use plant-derived polyphenols harboring a catechol group (Luscher et al. 2022). These compounds are intermediates of lignin biosynthesis, can bind iron, and are taken up via the promiscuous PiuA and PirA TonB-dependent transporters.

Finally, *Pseudomonas* spp. can also tap iron sources from animal hosts (Fig. 1f). The best studied systems involve the hemin (Phu) and heme (Has) uptake pathways of *P. aeruginosa* (Ochsner et al. 2000; Cornelis and Dingemans 2013; Smith and Wilks 2015). Recently, a third system (Hxu) has been described, that is involved in heme sensing and works in concert with the Has system (Otero-Asman et al. 2019). *P. aeruginosa* is an opportunistic pathogen of vertebrates and it is therefore straightforward to understand why it has evolved mechanism to acquire iron from the heme group that is part of hemoglobin (Marvig et al. 2014; Richard et al. 2019). While *phuR* codes for an outer-membrane transporter that is able to directly take up heme from the environment, *hasA* encodes a hemophore protein that is secreted to scavenge heme and to bring it back to the cell via the specific transporter HasR. Across *Pseudomonas* spp. it seems that the Phu-system is more common than the Has-system, with the latter being more restricted to pathogenic species (Cornelis and Bodilis 2009). Recently, it was discovered that *P. aeruginosa* can also capitalize on mammalian catecholamine neurotransmitters, molecules that chelate iron, and are taken up via the promiscuous PiuA and PirA TonB-dependent transporters (Perraud et al. 2022).

### The ecology and evolution of iron acquisition strategies

In the above sections, we have learnt that the iron acquisition repertoire of *Pseudomonas* spp. is extremely diverse. Why is this so? Why have species not evolved a single-best strategy—e.g. a siderophore with high iron affinity—to scavenge iron? This question can only be answered when adopting an eco-evolutionary perspective. We must not only describe how bacteria can mechanistically access iron, but also understand the ways through which organisms compete with one another for this essential nutrient at the community level. Competition is important to consider because it is recognized as one of the main drivers of evolutionary diversification (Day and Young 2004; Aristide and Morlon 2019). Tackling eco-evolutionary aspects is the goal of this section. I will start with simple communities and then gradually work towards more complex consortia with the aim to understand how the biotic environment could have influenced the evolution of iron acquisition strategies in *Pseudomonas* spp. At this stage, I must emphasize that it is often difficult to firmly conclude why exactly a certain mechanism has evolved and what the current and past selection pressures were. That is why several of the scenarios presented below must be understood as hypotheses, still to be scrutinized by future research.

#### Cooperative iron acquisition in clonal communities

**Fig. 4:**
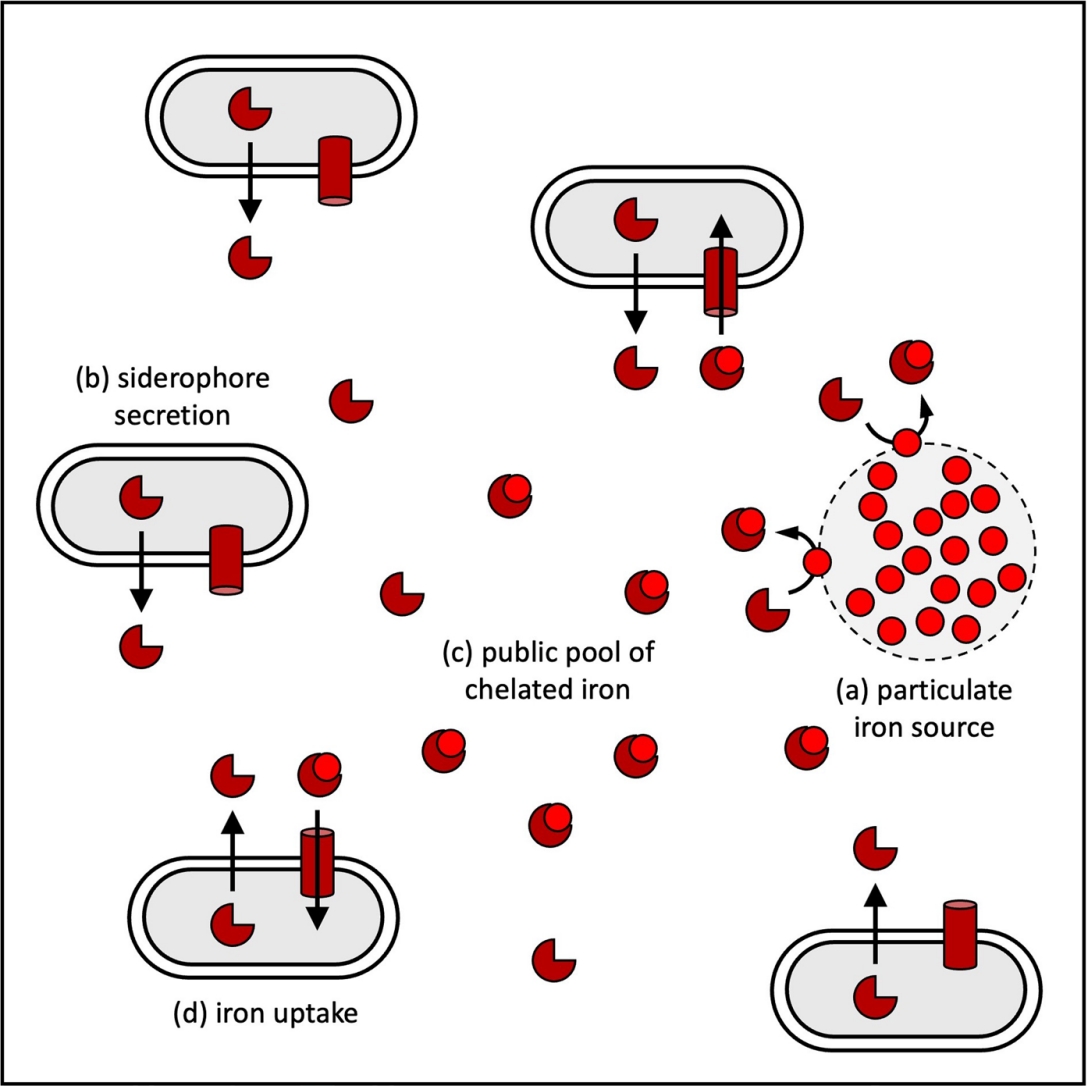
Siderophores as public goods in clonal populations. The depicted scenario shows a case where there is a single particulate source of iron (light red dots in grey circle) (**a**). All clonal bacteria secrete siderophores (dark red three-quarter circles) into the environment (**b**). The siderophores scavenge iron from the particulate source, creating a public pool of ferri-siderophores (**c**). All group members have the opportunity to take up ferri-siderophores from this public pool for their own use (**d**)

In clonal communities, the evolutionary interests of individuals are aligned (West et al. 2006). This means that any strategy that maximizes the reproductive output of the group should be selected for. Clonality and thus conditions of high genetic relatedness are conducive for the evolution of cooperation, where cooperative individuals help others to reproduce or divide (in the case of bacteria) (West et al. 2006). The production of costly siderophores has received a lot of attention in this the context, because siderophores are secreted and the scavenged iron can be taken up as public good by individuals other than the producers (Fig. 4) (Kramer et al. 2020). The process of siderophore secretion and sharing among clonal cells has been particularly well studied in *P. aeruginosa* (Griffin et al. 2004; Buckling et al. 2007; Kümmerli and Ross-Gillespie 2014). In this species, the siderophore production cost that accrues to individuals and the benefit that it generates for the group has been demonstrated for pyoverdine and pyochelin (Ross-Gillespie et al. 2015). Cooperative sharing of pyoverdine has also been studied in *P. fluorescens* (Zhang and Rainey 2013), *P. putida* (Becker et al. 2018) and environmental *Pseudomonas* spp. (Bruce et al. 2017; Butaitė et al. 2017; Butaitė et al. 2018). Overall, this body of work has revealed that the level and success of pyoverdine sharing depends on a range of factors, including population density (Ross-Gillespie et al. 2009), environmental viscosity (Kümmerli et al. 2009), distances between cells (Julou et al. 2013; Weigert and Kümmerli 2017), molecule diffusion (Weigert and Kümmerli 2017), resource availability and distribution (Brockhurst et al. 2008; Sexton and Schuster 2017; Stilwell et al. 2020). Despite the demonstrated cooperative benefits, it remains unclear whether siderophore secretion has indeed evolved because of its cooperative benefits (Völker and Wolf-Gladrow 1999; Driscoll and Pepper 2010; Zhang and Rainey 2013; Kramer et al. 2020). Theoretical work suggests that the secretion of diffusible siderophores has initially evolved because it reflects the most efficient way to bring iron into solution from particulate (i.e. clumped) stocks (Leventhal et al. 2019). Only later, selection for optimal levels of siderophore secretion and cooperative sharing might have been selected for. Such optimization strategies could, for example, involve the evolution of high pyoverdine durability and a recycling mechanism that allows the repeated use of the same pool of molecules within the community (Imperi et al. 2009; Kümmerli and Brown 2010). Overall, it was most likely a combination of abiotic environmental and social cooperative factors that had promoted the evolution of highly diffusible and shareable siderophores (Kümmerli et al. 2014).

#### Conflicts over iron acquisition between *Pseudomonas* strains and species

While the clonal scenario can explain the benefit of pyoverdine sharing, it cannot reveal why there are so many different variants of this molecule and its cognate transporter across *Pseudomonas* spp. (Meyer et al. 2008; Bodilis et al. 2009). While dozens of pyoverdine variants have been described worldwide, it turned out that the diversity also occurs at a very local ecological scale, among co-isolated strains from soil and freshwater habitats (Butaitė et al. 2017; Rehm et al. 2022). What factors can select for this diversity and why is it maintained over evolutionary time scales (Smith et al. 2005)? The most likely answer is not cooperation but competition.

**Fig. 5:**
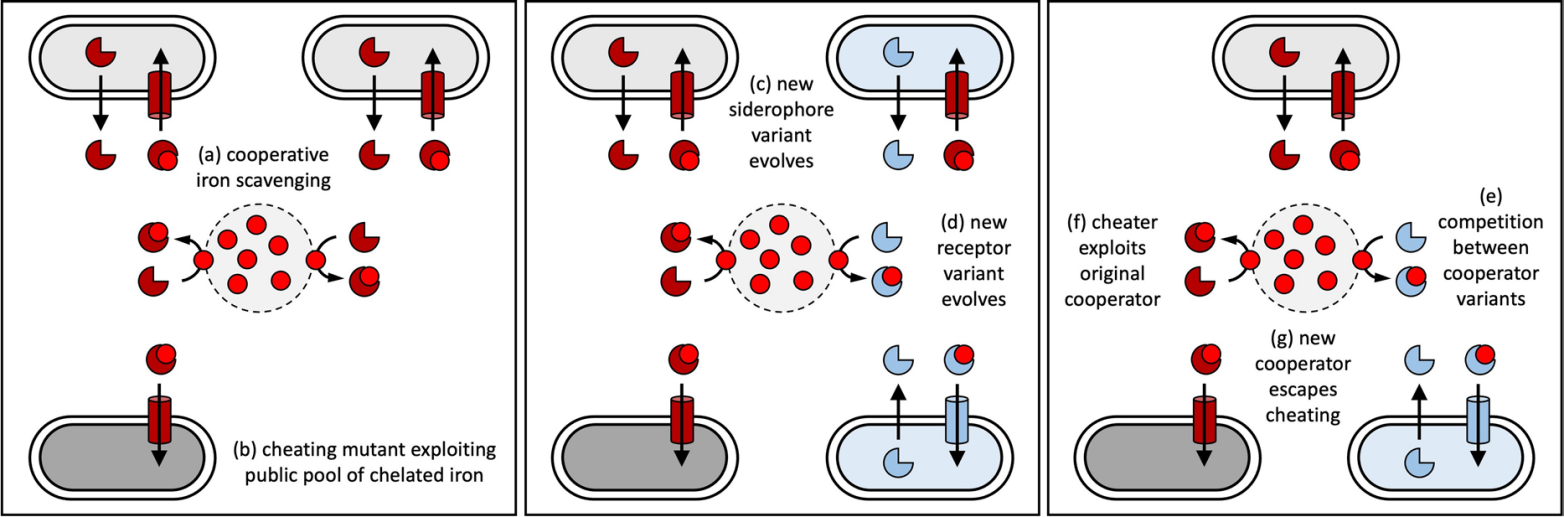
Cheating and how it could select for pyoverdine diversity. Left panel: Cooperative iron scavenging (**a**), as explained in Fig. 4, can select for mutants that no longer contribute to pyoverdine production (dark grey cells), but keep the transporter to access the public pool of ferri-pyoverdines created by producers (**b**). *Middle panel*: cheating could select for producer mutants (blue cells) that make a structurally different pyoverdine variant (blue three-quarter circle) (**c**). In the initial phase, this producer mutant makes a pyoverdine that no one can use, and it thus still relies on the original ferri-pyoverdine (red three-quarter circle) for iron scavenging. In a next step, the transporter mutates in the producer mutant to allow selective uptake of the novel pyoverdine variant (**d**). Right panel: pyoverdine and strain diversity can be maintained in the population when the cheater outcompetes the original cooperator (**e**), the new producer mutant is resistant to cheating (**f**) yet loses in competition against the original pyoverdine producer (**g**)

One type of competition occurs when the availability of secreted pyoverdine in the environment selects for non-producers that no longer invest in this costly molecule, but still express the transporter for uptake (Fig. 5a and b) (Ghoul et al. 2014; Smith and Schuster 2019). Such non-producers (often called cheaters) arise spontaneously in experimentally evolving lab cultures (Jiricny et al. 2010; Dumas and Kümmerli 2012) and have been isolated from the environment (Bruce et al. 2017; Butaitė et al. 2018) and chronic infections (De Vos et al. 2001; Jiricny et al. 2014; Andersen et al. 2015). Under conditions of high molecule diffusion, non-producers can become dominant in a population (Ross-Gillespie et al. 2007), and because they do not contribute to pyoverdine production, they can become a burden by compromising group productivity or even driving group extinction (Kümmerli et al. 2015). It was proposed that it is this cheater burden that can select for mutants with altered pyoverdine types, through mutations in one of the pyoverdine synthesis enzymes (Fig. 5c) (Smith et al. 2005; Lee et al. 2012; Figueiredo et al. 2021). For example, a mutated enzyme could build in a different amino acid into the pyoverdine peptide backbone, such that the pyoverdine is no longer compatible with the transporter of the non-producer. In a second step, the cognate transporter of the mutant must also change to gain optimal binding affinity for the novel pyoverdine variant produced (Fig. 5d). Once there are two types of pyoverdines in a population there will be competition between the two producers for access to iron (Fig. 5e) (Lee et al. 2012; Inglis et al. 2016; Stilwell et al. 2018). If the novel pyoverdine type cannot be exploited by cheaters but has lower affinity to iron than the original pyoverdine then a stable community can arise, where different pyoverdine strategies are maintained over time (Fig. 5e–g) (Inglis et al. 2016). In such a community, (i) cheaters win against the original pyoverdine producer, (ii) the original pyoverdine producer wins against the novel pyoverdine producer, and (iii) the novel pyoverdine producer wins against the cheater. Thus, the three strains chase each other in circels with no overall winner, a pattern known as rock-paper-scissors dynamic (Kerr et al. 2002).

While cheating and producer competition are intuitive to understand and could be plausible drivers of pyoverdine diversity, there are a number of unsolved aspects. The most obvious one is that at least two mutations are required within the same individual to (i) change the pyoverdine synthesis cascade and (ii) alter the transporter to optimize affinity for the novel pyoverdine. It is very unlikely that these two mutations happen at the same time. They most likely happen sequentially, which means that any new pyoverdine variant is initially produced by a mutant that is compromised at taking up its own siderophore. Although there is theoretical work showing that such mutants can survive in populations until the point where their transporter adapts to their new pyoverdine variant (Lee et al. 2012), an experimental demonstration of this process turned out to be difficult (Figueiredo et al. 2021). Furthermore, there might be alternative selection pressures that act on pyoverdine diversity. For example, pyoverdine transporters are entry points for toxins (pyocins) and potentially also for phages into the bacterial cell (Baysse et al. 1999; Smith et al. 2005; Denayer et al. 2007; Elfarash et al. 2014). Thus, there could be selection to modify the transporter to prevent such attacks, which in turn could favor the respective changes in the pyoverdine synthesis machinery. This scenario seems also plausible but remains untested so far. Finally, there is only a finite number of potent pyoverdines and transporter variants available. Given that all pyoverdines and transporters belong to the same families of molecules and proteins, respectively, we would expect that specificity has its limits and that any given transporter can take up a range of pyoverdine variants, although with varying efficacies (Meyer et al. 1997; Greenwald et al. 2009; Bruce et al. 2017; Butaitė et al. 2021).

To sum up, there is clear evidence for diversifying selection for both pyoverdines and transporters in *Pseudomonas* communities although the drivers of diversification need yet to be determined. Clear is also that the diversification spurs interaction networks among genetically diverse strains that entail pyoverdine cheating/exploitation, mutual cross-use and sharing among clonemates (Bruce et al. 2017; Butaitė et al. 2021; Figueiredo et al. 2022), with these cooperative and competitive interactions having the potential to foster community stability (Inglis et al. 2016).

#### Competition over iron acquisition between different bacterial species

**Fig. 6:**
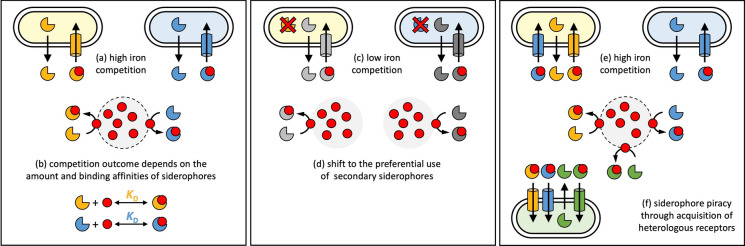
Evolutionary scenarios in inter-species competition for iron. Left panel: In a scenario where two bacterial species compete for the same limited stock of iron (**a**), the outcome of competition depends on the relative iron binding affinities (*K*_D_) of the two competing siderophores (orange vs. blue three-quarter circles) and the relative amounts of siderophores produced (**b**). Competition could thus select for higher siderophore production levels and for siderophores with increased binding affinities. Middle panel: under conditions of increased iron availability, where species do not compete for the same iron stocks (**c**), bacteria might reduce investment into their expensive primary siderophores (colored three-quarter circles) and shift to the production and use of their less expensive secondary siderophores (grey three-quarter circles) (**d**). This scenario could explain the evolutionary maintenance of low-iron affinity secondary siderophores. Right panel: A scenario is shown where competing species use their unique primary siderophore (colored three-quarter circles) to compete for the same limited stock of iron (**e**). This scenario could select for the acquisition of transporters from competitors (probably via horizontal gene transfer) to pirate on each other’s ferri-siderophores **(f)**

In this section, we move one level up and consider interactions of *Pseudomonas* spp. with species from other taxa. Members of other taxa typically produce structurally different siderophores, like enterobactin, staphyloferrin, ornibactin, aerobactin and many others (Hider and Kong 2010). Under iron-limited conditions, the secreted siderophores enter in direct competition with pyoverdine and any other siderophore produced within the community (Harrison et al. 2008; Niehus et al. 2017; Leinweber et al. 2018; Gu et al. 2020a; Sathe and Kümmerli 2020). The equilibrium condition of how much iron is bound to pyoverdine vs. these heterologous siderophores depends on the concentrations of the competing siderophores in the environment and their relative iron binding affinities (Fig. 6a and b). One conceivable consequence of this form of inter-species competition is that it selects for higher levels of siderophore production and for siderophores with increased iron affinity to push the chemical equilibrium of bound iron towards the focal species. If this selection pressure operates in both the competing species, it could lead to an evolutionary arms race and the selection for siderophores with ever increasing iron affinities. Arms races could explain the incredibly high iron affinities of certain siderophores like enterobactin (K_a_ = 10^52^ M^−1^) (Raymond et al. 2003), but also pyoverdine (K_a_ = 10^32^ M^−1^) (Albrecht-Gary et al. 1994).

But not all siderophores have such high iron affinity. For instance, the iron affinity of pyochelin is lower (ethanol (Cox and Graham 1979): K_a_ = 2*10^5^ M^−1^; aqueous solution (Brandel et al. 2012): K_a_ = 10^28^ M^−2^). How can we explain the evolution and selective maintenance of lower affinity siderophores? As we have learnt above, pyochelin is a secondary siderophore, preferentially produced under moderate iron limitation (Cornelis and Dingemans 2013; Dumas et al. 2013; Mridha and Kümmerli 2022). A plausible hypothesis for its evolutionary maintenance is that pyochelin is cheaper to produce and sufficient for iron scavenging under moderate iron limitations, where between-species competition for iron is less severe and a low-affinity siderophore would do the job (Fig. 6c and d) (Dumas et al. 2013). This hypothesis clearly needs more detailed investigations, and it would be important to see whether it also applies to other bacterial species with secondary siderophores.

Finally, there is a second way of how bacteria in general, and *Pseudomonas* spp. in particular, can get an edge in the competitive race for iron. This way operates via the ability to tap the siderophore pool of the competing species, a strategy often referred to as ‘siderophore piracy’ (Fig. 6e) (Traxler et al. 2012; Galet et al. 2015; Perraud et al. 2020). Like cheating in the context of within-species competition, siderophore piracy is enabled when a species possesses transporters of heterologous siderophores produced by its competitors. Indeed, *Pseudomonas* spp. possess transporters for several heterologous siderophores like enterobactin and desferrioxamines. But how were these transporters acquired in the evolutionary past? We do not know for sure. The most likely explanation is that heterologous siderophore transporters can be obtained via horizontal gene transfer from the competing species. Subsequent patterns of mutation and selection could change the specificity of the transporter towards certain heterologous siderophores or lead to the evolution of a promiscuous transporter that are able to take up several different types of siderophores (Cuiv et al. 2006; Lee et al. 2012; Chan and Burrows 2022).

Siderophore piracy seems also to play a prominent role in competition between *Pseudomonas* spp. themselves, as it turned out that *P. aeruginosa* (Ghysels et al. 2004), *P. fluorescens* (Moon et al. 2008) and *P. protegens* (Sexton et al. 2017) but also many environmental species (Ye et al. 2014; Butaitė et al. 2017) not only possess a cognate transporter for their own ferri-pyoverdine, but also multiple copies of pyoverdine transporters that can take up pyoverdine variants produced by other species and strains (Butaitė et al. 2018). Within *Pseudomonas* spp., horizontal gene transfer and gene duplication could both result in increased copy numbers of pyoverdine transporters within a given genome. Especially gene duplication could be a powerful mechanism, as the paralogues could evolve independently: the native transporter gene can be optimized for the self-produced (ferri-)pyoverdine, while the duplicated gene could adapt and be optimized for the uptake of heterologous (ferri-)pyoverdines. It is yet unclear how siderophore piracy affects community stability. However, principles as the rock-paper-scissors scenario could also apply here (Inglis et al. 2016), namely that piracy leads to complex social interaction networks among species that stabilize community composition (Morris 2015).

#### Iron competition between Pseudomonas spp. and eukaryotes

As discussed above, many *Pseudomonas* spp. possess transporters to take up iron-chelating molecules produced by fungi, plants and vertebrates. From an ecological perspective, this observation is conceivable as *Pseudomonas* spp. can thrive in many different environments, including freshwater habitats, the rhizosphere, and animal hosts (Silby et al. 2011; Moradali et al. 2017). However, more intriguing is the question about how *Pseudomonas* spp. acquired these uptake mechanisms during their evolution. In soil, there is close interaction between bacteria and fungi (Deveau et al. 2018), and horizontal gene transfer is known to occur across the kingdom level (Schmitt and Lumbsch 2009; Li et al. 2018). While horizontal gene transfer could represent a way of how *Pseudomonas* spp. have acquired fungal siderophore transporters, an alternative explanation is that mutational modifications of existing bacterial siderophore transporters has increased promiscuity, allowing the uptake of fungal siderophores.

The situation seems different for interactions with plants, for which beneficial interactions with *Pseudomonas* spp. have been repeatedly reported (Kloepper et al. 1980; Raaijmakers et al. 1995; Mercado-Blanco and Bakker 2007; Passera et al. 2019). Specifically, pyoverdines were shown to suppress the growth of plant pathogens (Gu et al. 2020b), and models suggest that plants recruit beneficial *Pseudomonas* spp. to their rhizosphere. One possible way of recruitment could operate via plant root exudates containing ferri-citrate, which are then taken up by *Pseudomonas* spp. that possess the ferri-citrate transporter. However, these assertions remain speculative and still need to be tested.

Little is known on the evolutionary origin of the various heme-derived iron uptake systems (Fig. 1e and f). As mentioned before, the more sophisticated Has-system seems to be more prevalent among pathogenic species (Cornelis and Bodilis 2009), which indicates that it could be a host-specific adaptation. It was further shown that the Phu-system (heme uptake) in *P. aeruginosa* is under selection for improved performance in chronic infections of cystic fibrosis patients (Marvig et al. 2014; Andersen et al. 2018), highlighting the importance of the acquisition of iron via heme. In contrast to plants, interactions between *Pseudomonas* spp. (particularly *P. aeruginosa*) and vertebrate hosts are clearly competitive by nature. This is demonstrated by the fact that hosts have evolved mechanisms to withhold iron from bacterial pathogens through the sequestration of transferrin and lactoferrin, proteins that bind iron with high affinity, and the production of siderocalin, a protein that binds and thereby curbs siderophore activity (Cassat and Skaar 2013; Becker and Skaar 2014).

## Conclusion

In this review, I aimed to provide an overview on the versatile iron acquisition mechanisms and their evolution in *Pseudomonas* spp. While we have a very detailed understanding of the mechanistic basis of iron acquisition, our knowledge on their evolutionary past and contemporary selection pressures is still incomplete. The eco-evolutionary scenarios elucidated in this review suggest that competition among *Pseudomonas* spp. and competition with other bacterial taxa, fungi and animal hosts played a prominent role in shaping the diversification of iron-uptake strategies. While I focused on *Pseudomonas* spp. as one of the best studied taxa in the context of iron acquisition, the proposed concepts are applicable to all microorganisms. The concept that emerges from the integration of mechanistic, ecological, and evolutionary aspects is that competition for iron plays an important role in shaping interactions patterns among microbes in complex communities and might also be involved in determining community assembly and stability.
